# Utilization of nanotechnology and experimental design in the development and optimization of a posaconazole‒calendula oil nanoemulgel for the treatment of mouth disorders

**DOI:** 10.3389/fphar.2024.1347551

**Published:** 2024-02-15

**Authors:** Mohammed Alissa, Ahmed Hjazi, Ghadah S. Abusalim, Ghfren S. Aloraini, Suad A. Alghamdi, Nahed S. Alharthi, Waleed Y. Rizg, Khaled M. Hosny, Nada Binmadi

**Affiliations:** ^1^ Department of Medical Laboratory, College of Applied Medical Sciences, Prince Sattam Bin Abdulaziz University, Al-Kharj, Saudi Arabia; ^2^ Department of Pharmaceutics, Faculty of Pharmacy, King Abdulaziz University, Jeddah, Saudi Arabia; ^3^ Center of Innovation in Personalized Medicine (CIPM), 3D Bioprinting Unit, King Abdulaziz University, Jeddah, Saudi Arabia; ^4^ Department of Pharmaceutics and Industrial Pharmacy, Faculty of Pharmacy, Beni-Suef University, Beni-Suef, Egypt; ^5^ Department of Oral Diagnostic Sciences, Faculty of Dentistry, King Abdulaziz University, Jeddah, Saudi Arabia

**Keywords:** ulcer index, posaconazole, gingivitis, nanoemulsions, box-behnken design

## Abstract

**Introduction:** Essential oil‒based nanoemulsions (NEs) are the subjects of extensive investigation due to their potential to address a variety of oral health issues. NEs are delivery systems that improve lipid medicine solubility and distribution to intended sites. The goal of the current study was to create and enhance a self-nanoemulsifying drug delivery paradigm based on calendula oil (CO) and decorated with chitosan (CS) that could deliver posaconazole (PSZ) for the treatment of gingivitis.

**Method:** Employing a response-surface Box‒Behnken design, PSZ-CO-CS NEs were created with varying amounts of PSZ (10, 15, and 20 mg), percentages of CO (6%, 12%, and 18%), and percentages of CS (0.5%, 1.5%, and 2.5%).

**Results and conclusion:** The optimized formulation resulted in a 22-mm bacterial growth suppression zone, 25-mm fungal growth inhibition zone, droplet sizes of 110 nm, and a viscosity of 750 centipoise (cP). Using the appropriate design, the ideal formulation was produced; it contained 20 mg of PSZ, 18% of CO, and 1.35% of CS. Furthermore, the optimal formulation had a more controlled drug release, larger inhibition zones of bacterial and fungal growth, and desirable rheologic properties. Additionally, the optimized formulation substantially lowered the ulcer index in rats when tested against other formulations. Thus, this investigation showed that PSZ-CO-CS NEs could provide efficient protection against microbially induced gingivitis.

## 1 Introduction

The human oral cavity continues to be the primary entry point for a diverse range of environmental microorganisms, including viruses, fungi, and protozoa, but predominantly bacteria (Dewhirst et al., 2010). There are around 600 bacterial species (Dewhirst et al., 2010) and 100 fungal species (Ghannoum et al., 2010) in this diverse microbiome. *Candida albicans*‒caused candidosis, often known as candidiasis, is thought to be the most prevalent fungal infection in the oral cavity. However, non-*albicans Candida* species are also being seen as causes of candidiasis in some patient populations (Pfaller et al., 1995). *Candida* is well known for its induction of polymicrobial illnesses in humans as it can produce multispecies biofilms (Samaranayake et al., 1987). The tissues that protect and sustain the teeth make up the periodontium, which is considered the major site of periodontal disease, also known as periodontitis, a chronic inflammatory condition (Darveau, 2010). It is brought on by a synergistic population of bacteria found in tooth plaque, with keystone pathogens such as *Porphyromonas gingivalis* initiating a breakdown of tissue homeostasis (Hajishengallis et al., 2012). A variety of microbes, including bacteria, fungi, and, potentially, viruses, make up this tooth “plaque biofilm” (Offenbacher, 1996). A number of investigations have found that unlike people with a healthy periodontium, patients with chronic periodontitis exhibit higher levels of subgingival colonization by yeasts, specifically *C. albicans* (Canabarro et al., 2013).

Bacterial infection is the most prevalent cause of gingivitis, an inflammatory disease of the gingival tissue limited to the gingival epithelium and surrounding connective soft tissues (Marchesan et al., 2020). The most prevalent periodontal disease is thought to be gingivitis. There are several types of gingivitis, and they are distinguished by their etiology, severity and length of infection, and clinical presentation. The most common kind is thought to be the chronic one brought on by plaque. Clinically, the gingival tissues exhibit redness, swelling, a glossy surface, and mild bleeding when prodded and cause discomfort to the patient. Since gingivitis typically causes minimal pain and infrequently results in spontaneous bleeding, many patients do not acknowledge the illness or seek treatment for it (Trombelli et al., 2018).

Derived from itraconazole, posaconazole (PSZ) is a systemic triazole antifungal medication that has the same mechanism of action as comparable azole analogues (Andes et al., 2004). Like various azole derivatives, PSZ prevents the production of ergosterol, a crucial component of fungal cell membranes, by blocking the activity of lanosterol 14α-demethylase. PSZ’s antifungal action is thought to be caused by the depletion of ergosterol in the cell membrane and the buildup of methylated sterol precursors, which degrade the fungal cell membrane’s structure and function (Petraitiene et al., 2001). In *in-vitro* conditions, PSZ exhibits broad effectiveness against most vulnerable pathogenic yeasts and molds, including species such as *Aspergillus* and *Candida*, as well as less prevalent pathogens, including several species of *Fusarium* and *Mucorales* (Chen et al., 2020). PSZ’s poor solubility makes its distribution challenging, despite its broad-spectrum action (Sousa et al., 2019). PSZ is highly permeable and poorly soluble, placing it in Biopharmaceutical Classification System class II. Its low oral bioavailability, which ranges from 8% to 47% owing to its high lipophilicity, means that several methods are needed to boost its dissolution and bioavailability (Hens et al., 2018). Nanotechnology-based drug delivery systems are increasingly thought to have a positive impact on the active agents’ solubility, activity, and bioavailability (Alhakamy et al., 2022; Salem et al., 2022; Abdulaal et al., 2023; Salem et al., 2023). Therefore, employing nanosized systems such as nanoemulsions (NEs) and nanoparticles could be a promising solution. Sundry investigations have reported quite similar findings (Ali et al., 2021; Elkomy et al., 2021; Rizg et al., 2021; Hussein et al., 2022).

*Calendula officinalis* L., family Compositae, is an annual herb of Mediterranean origin. It is thought to have arrived in England in the thirteenth century (Parente et al., 2012). Its use for inflammatory lesions in the oral and pharyngeal mucosae was recommended by German sanitary authorities (Re et al., 2009). Triterpenes, alcohol triterpenes, saponins, fatty acids and their esters, carotenoids, coumarins, flavonoids, and essential oils are the primary chemical constituents of *C. officinalis* flowers (Neukirch et al., 2004; Shahane et al., 2023). The animal model of ear edema induction employing croton oil and another model of paw edema initiation via carrageenan have been used to assess the anti-inflammatory action of *C. officinalis* flowers cultivated in Europe and Asia (Patrick et al., 1996; Preethi et al., 2009; Beg et al., 2011).

An NE is a sophisticated delivery system that can be used to improve characteristics such as stability and solubility (Barradas and Silva, 2021). NEs have been extensively researched as drug delivery vehicles. They are composed of nanosized oil droplets stabilized by a combination of co-surfactants and surfactants (Hosny et al., 2020). The main benefit of NEs is their existence as nanometric-sized globules in the oil phase. Consequently, using a calendula oil (CO)‒based NE in the treatment of oral microbiota is a potentially beneficial strategy (Aithal et al., 2020). The essential oil is more strategically delivered to the target location in the oral cavity when it is used in an oral gel formulation. Consequently, it would be more efficient to convert an NE into a gel formulation (Aslani et al., 2016).

A thickening or gelling ingredient is added to an NE to create an NE gel. Such a gel has an improved length of residence in the oral cavity and boosts the potential for sustained drug release (Mahmoud et al., 2010; Aslani et al., 2016). Previous research has shown that a nanoemulgel increases the solubility of medications and enhances their permeability and efficiency in both *in-vitro* and *in-vivo* settings. Because of their ability to alter the appearance, flow behavior, and greasiness of a drug delivery system, nanoemulgels have a longer residence period in the oral mucosa and may be easily wiped off as and when needed (Mahmoud et al., 2010). Nanoemulgels have experienced a notable surge in demand and application owing to their enhanced thixotropic and non-specking properties, long shelf life, emollient characteristics, ability to deliver hydrophobic medications, and spreadability (Alexander et al., 2013).

To provide a longer retention time and stronger bond with the nasal epithelium, chitosan (CS), a naturally occurring, positively charged, biodegradable polymeric material, has been used as a mucoadhesive and penetration-enhancing agent because the nasal mucosa is negatively charged (Nafee et al., 2007; Abul Kalam et al., 2016). The enzymatic conversion of CS into its simple, non-toxic components was performed. Lysozyme, a non-specific protease present in all mammalian tissues, is principally responsible for the *in-vivo* breakdown of CS’s components. Non-toxic oligosaccharides that can be eliminated or integrated into glycosaminoglycans and glycoproteins were produced as a result of this transformation (Szymańska and Winnicka, 2015).

Statistical techniques are employed in experimental design approaches and can be applied in the process of optimizing a new formulation. Their application in the formulation and refinement of pharmaceuticals has been demonstrated (Politis et al., 2017; Hosny et al., 2019). Finding the ideal formulation requires many fewer trials and much less work when statistical optimization designs are used. Moreover, the results are easily evaluable and readily generalized to many formulation factors (Yang et al., 2014).

Based on the aforementioned data from the literature, the goal of the current study was to create a nanoemulgel containing PSZ and CO and assess its ulcer index and antibacterial and antifungal properties. Using DesignExpert software, the Box‒Behnken statistical design was used to optimize the nanoemulgel. To our knowledge, our study was the first to investigate the results of PSZ combined with CO used to treat certain illnesses of the oral cavity, such as gingivitis and oral thrush.

## 2 Materials and methods

### 2.1 Materials

Posaconazole (PSZ) was purchased from Merck Chemicals, located in Darmstadt, Germany. The Saudi-Indica Natura Company (Jeddah, Saudi Arabia) provided calendula oil (CO) (essential oil prepared through hydrodistillation). The following products were purchased from Sigma (St. Louis, Missouri, United States): Nonoxynol-10, sodium deoxycholate, Polyoxyl 20 cetostearyl ether, diacetylated monoglycerides, sucrose stearate, Tween 20, glycerol propylene glycol, glycofurol, butylene glycol chitosan, and PEG 200. Gattefosse (Saint-Priest, France) kindly supplied Labrasol, Maisine CC, and Transcutol. All of the other substances used in this experiment were analytical in nature. The water used in the trials was purified.

### 2.2 Methods

#### 2.2.1 Assessment of posaconazole’s solubility in different surfactants and co-surfactants

To screw-capped glass vials that held an established amount of 2 mL of each tested material, an excess amount of PSZ was added. The surfactants used were Nonoxynol-10, Labrasol, Polyoxyl 20 cetostearyl ether, diacetylated monoglycerides, Tween 20, and sucrose stearate, and the employed co-surfactants were butylene glycol, sodium deoxycholate, Transcutol HP, propylene glycol, glycofurol, PEG 200, Maisine CC, and glycerol.

The substances were mixed using a vortex mixer and stored for 72 h at 25°C ± 0.5°C in a temperature-controlled shaking water bath. One milliliter of the mixture was centrifuged for 15 min at 14,000 rpm after equilibrium was reached. For an additional 15 min, the clear supernatant solution was centrifuged. To find the amount of PSZ at λ_max_ 260 nm, the contents of each vial were properly diluted with methanol and then measured by spectrophotometry. Each experiment was run in triplicate, and the mean plus or minus the standard deviation (SD) was given in milligrams per milliliter (Rizg et al., 2021).

#### 2.2.2 Pseudoternary phase diagrams

Pseudoternary phase diagrams focused on the blend of surfactants and co-surfactants (S_mix_), deionized water, and CO. Aqueous titration was used to produce NEs from the oil, S_mix_, and deionized water. Varying ratios of the selected surfactant to co-surfactant (e.g., 1:1, 1:2, 1:3, 2:1, 3:1) were employed. Various weight ratios of CO and the S_mix_ were blended in accordance with each phase diagram, and these mixes were subsequently gradually adjusted with the aqueous phase (deionized water). This was done without heating the mixes, and a soft magnetic stirrer was used. The level of water at which transparent-to-turbid shifts began to occur was considered the titration’s endpoint (Hosny et al., 2021a).

#### 2.2.3 Fabrication and optimization of self-nanoemulsion formulations using an experimental design approach

Design-Expert (version 12.0.6.0; Stat-Ease, Inc., Minneapolis, Minnesota, United States) software was utilized to build self-nanoemulsifying drug delivery system (SNEDDS) formulations. The response surface Box‒Behnken design was used to examine the effects of the independent variables on the *in-vitro* characteristics and *in-vivo* efficacy metrics. The independent variables were the amount of PSZ (A) (amount of mg of PSZ in 1 gm of nanoemulsion), the percentage of CO (B) (percentage of CO in 1 gm of nanoemulsion. i.e. 18% of CO mean that each 1 gm of nanoemulsion contain 0.18 gm of CO), and the percentage of chitosan (CS) (percentage of chitosan in aqueous phase used in the formulation of nanoemulsion. i.e. 1.5% pf CS mean that the used aqueous phase during nanoemulsion preparation is not pure distilled water, it was CS solution prepared by concentration of 1.5% chitosan in water). The investigated dependent responses were the average vesicle size (Y_1_), viscosity (Y_2_), inhibition zone against *Candida* (Y_3_), and antimicrobial activity against streptococci (Y_4_). The selected responses and their levels and independent variables, along with their constraints, are displayed in Table 1.

**TABLE 1:** Three-factor, three-level Box‒Behnken statistical design: independent variables at different levels.

Independent variable	Levels		
	Low (−1)	Medium (0)	High (1)
A = Amount of PSZ (mg)	10	15	20
B = Percentage of CO (%)	6	12	18
C = Percentage of CS (%)	0.5	1.5	2.5
Dependent variables		Constrains	
Y_1_ = Vesicle size		Maximize	
Y_2_ = Viscosity		Maximize	
Y_3_ = Inhibition zone against *Candida*		Maximize	
Y_4_ = Antimicrobial activity against streptococci		Maximize	

#### 2.2.4 Preparation of PSZ-CO-CS self-nanoemulsion

The procedure used for the preparation of a PSZ-loaded NE formulation had two stages (Hosny et al., 2021a). The first step involved creating the basic SNEDDS. In accordance with the design, a constant amount of the S_mix_ 1:2 was mixed with 6%, 12%, and 18% CO. In the second step, the solid ingredient (PSZ) was mixed with the simple SNEDDS in amounts of 10, 15, or 20 mg in line with the design displayed in Table 1 (Hosny et al., 2021a). Finally, the primary NE was prepared by mixing 1 gm of PSZ-loaded SNEDDS with 9 mL volume of the aqueous dispersion of CS with different CS percentages as shown by the design in Table 1. It is noteworthy that before formulating the 20 formulations provided by the software, a preliminary study was done to test the maximum amount of posaconazole that 1 gm of nanoemulsion can carry and still measure in nano size range. The loading efficiency for 5 nanoemulsion differ in their composition (ratios of oil to surfactant and cosurfactant) were determined. Results indicated that the loading efficiency was <98% for all tested samples if the posaconazole used was less than 50 mg/g of nanoemulsion. In the study design as indicated in Table 1, the maximum amount of posaconazole used in all tested formulation in the design was 20 mg/gm. So no need to incorporate the loading efficiency as one of the dependent variable as it will be more than 98% for all formulations. Furthermore, The loading efficiency for the optimized formula obtained was measured, and its result was 100%.

#### 2.2.5 Characterization of the fabricated PSZ-CO-CS NE

##### 2.2.5.1 Droplet size

Using the Zetatrac device (Microtrac, Montgomeryville, Pennsylvania, United States), the vesicle size of the artificial PSZ-CO-CS NE formulations was calculated based on the formula produced by the statistical software. Eventually, 900 μL of purified water was used to dilute the drug-loaded NE (100 μL) formulation 10 times, and the mixture was vortexed. To determine the average vesicle size, each sample from each formulation batch was examined three times (Hosny et al., 2020).

##### 2.2.5.2 Viscosity

A Brookfield viscometer equipped with a spindle 52 was used to measure the viscosity of the manufactured PSZ-CO-CS NE formulations based on the combinations produced by the statistical software. One Gram of the corresponding samples was used to determine the related evaluation parameters. The experiment was conducted at the ambient temperature (25°C ± 1°C), and the recorded measurements over the preset shear rate of 2 s^-1^ were analyzed to determine the flow pattern of the produced samples (Mandal et al., 2012).

##### 2.2.5.3 Antifungal efficiency of various PSZ-CO-CS NE formulations

The disc diffusion technique was used to assess the PSZ-CO-CS NE formulation’s *in-vitro* antifungal effectiveness. To prepare sterile Petri dishes, Sabouraud dextrose agar medium (pH 6.3) was utilized. To inoculate the Petri dishes, the fungal strains of *C. albicans* (ATCC 10231, Oxoid Ltd., Basingstoke, England) and was also utilized. were cultured in normal saline. Sterile swabs were used to inoculate the Petri plates with the fungi after they had been submerged in the ready-made fungal suspension. The purpose of the Petri dishes was to create a cultivated lawn. After that, 100 µL of the resulting PSZ-CO-CS NE formulations was placed on sterile filter paper discs measuring 6 mm, which were then placed on the infected Petri dishes. After 48 h of incubation at 24°C ± 2°C, the Petri dishes’ zone of inhibition was determined using a ruler (Krishnamoorthy et al., 2021).

##### 2.2.5.4 Antibacterial activities of PSZ-CO-CS NEs

An analogous experiment utilizing an agar plate as the medium was conducted for streptococci. It is essential to note that there have been reports linking this bacterium to the common microbe that causes gingivitis. *S. mutans ATCC 25175* was the *streptococcus* species that was used; it was isolated from carious dentin. S. mutans is thought to be the most cariogenic of all oral bacteria and the main culprit in plaque development and the start of tooth caries. The zone of inhibition for each of the tested PSZ-CO-CS NE formulations was ascertained during this experiment.

After being removed from the cultivated plates, the streptococcal culture was kept alive in liquid culture media. An ultraviolet-visible spectrophotometer was used to evaluate the optical density of the culture at 550 nm after the cocci had grown sufficiently at 37°C. After diluting the growth fluid to an optical density of 0.3, the material was swabbed into the 20-mL chilled agar Petri dishes. Subsequently, wells of 12 mm in diameter were bored using a sterile punch borer into the infected plates to accommodate the loading of 100 µL of the formulation. After the Petri plates were incubated at 37°C for 18 h, the zone of inhibition created by the PSZ-CO-CS NE formulations was compared with the zone of inhibition brought about by the sterile water (negative control) and blank NE. Every experiment was carried out three times (Feng et al., 2022).

#### 2.2.6 Optimization of the derived PSZ-CO-CS NE formulations

All independent variables and their relationships had their F-ratio, *p*-value, and degrees of freedom calculated for the analysis of variance (ANOVA) of the derived models. Based on the findings, the responses were used to select the model that most closely matched the acquired data. A *p*-value of less than .05 especially indicated the significance of the examined model terms. Using the CV% data, correlation coefficients, predicted R^2^, and adjusted R^2^, the model’s fitness was further assessed. The optimal factors were selected based on the created PSZ-CO-CS NE’s ability to achieve the four dependent variable objectives: minimize the globule size and maximize the viscosity, inhibition zone against *Candida*, and antimicrobial activity against streptococci.

#### 2.2.7 Characterization of the developed PSZ-CO-CS NE

##### 2.2.7.1 Droplet size, polydispersity index, and zeta potential

The globule size, polydispersity index (PDI), and zeta potential of the optimized PSZ-CO-CS NE formulation were measured using the Malvern Zetasizer Nano ZSP (Malvern Panalytical Ltd., Malvern, Worcestershire, United Kingdom) instrument. The formulation was diluted with 900 µL of double-distilled water in a volumetric flask (Hosny et al., 2020).

##### 2.2.7.2 Antifungal studies for the optimized PSZ-CO-CS NE formulation

The optimized PSZ-CO-CS NE (F1), optimized formula without PSZ (F2), optimized formula with oleic acid in place of CO (F3), and oily dispersion of PSZ in CO (F4) were all subjected to an *in-vitro* antifungal efficacy test using the disc diffusion method, previously described in Section 2.2.5.3, against *C. albicans* (Krishnamoorthy et al., 2021).

##### 2.2.7.3 Antibacterial activities of the optimized PSZ-CO-CS NE formulation

An experiment similar to that described in Section 2.2.5.4 was performed for streptococci using the agar plate as the medium. During this experiment, the zones of inhibition were determined for the tested optimized PSZ-CO-CS NE (F1), optimized formula prepared without PSZ (F2), optimized formula prepared with oleic acid instead of CO (F3), and oily dispersion of PSZ in CO (F4) (Feng et al., 2022).

##### 2.2.7.4 Checking for stability using the freeze‒thaw cycle

For the developed, optimized PSZ-CO-CS NE formulation, three freeze‒thaw cycles of between −25°C and 25°C) were established, with storage at each temperature for 48 h. Particle size analysis was used each time to assess the size of the globule. The stability index was also established employing the following equation:

$$\text{Stability index}=\left(\left[\text{Initial size}-\text{Change in size}\right]/\text{Initial size}\right)\times 100$$ (1)

##### 2.2.7.5 Evaluation of the PSZ-CO-CS NE formulations’ rheologic characteristics

The rheological properties of the optimized PSZ-CO-CS NE formulation (F1), which was prepared with an aqueous phase of 1.35% CS solution, and the same PSZ-CO-CS NE formulation (F0) prepared with an aqueous phase of water instead of the CS solution were closely analyzed. A Brookfield viscometer with a spindle number of 52 was used to calculate the associated assessment parameters based on 1 g of the pertinent samples taken together. The study was carried out at 25°C ± 1°C, or room temperature. To ascertain the flow pattern of the produced samples, the recorded data were evaluated throughout predefined shear rate ranges (2, 10, 20, 30, 40, 50, and 60 s^-1^). As a result, the viscosity (cP), shear stress (dyne/cm^2^), and shear rate (sec^-1^) were plotted and verified. Finally, the degree of flow and pseudoplasticity (Farrow’s constant) at the maximum (max) and minimum (min) rates of shear were determined applying Farrow’s equation (Feng et al., 2022):

LogG=N.LogF –Logղ (2)

where *G* is the shear rate, *F* is the shear stress, *N* is the Farrow’s constant, and ƞ is the viscosity.

##### 2.2.7.6 *In vitro* release of the PSZ-CO-CS NE formulations

The *in-vitro* release behavior of the formulations was evaluated using a modified version of a previously published method (Abdelrahman et al., 2015). The *in-vitro* release profiles of the PSZ from the PSZ aqueous dispersion (V1), the optimized PSZ-CO-CS NE (V2), and the optimized formulation produced without CS (V3) were examined using a type I USP dissolution apparatus. Instead of a basket, the gadget features cylindrical tubes that are 10 cm long and 2.7 cm in diameter placed within. A semipermeable dialysis membrane with molecular weight cut off 12,000 kDa (Merck, Germany) was used to seal the tube’s bottom. The dialysis membrane was stimulated by immersing it for 1 min in a solution of 0.3% (W/V) of sodium sulfide at 800°C before it was attached to the glass tube.

Before being employed, the membrane was stored for 2 h in the refrigerator with a PBS of pH 6.8. Each study sample contained 20 mg of PSZ, or 1 mL of each formulation. A physiologic temperature of 37 ± 0.5°C was maintained while 250 mL of PBS (pH 6.8) was added to the vessel and swirled at 50 rpm to determine the PSZ release profile of the formulations. The PBS (pH 6.8) was prepared using anhydrous sodium dihydrogen phosphate (KH_2_PO4), anhydrous sodium hydrogen phosphate (Na_2_HPO4), sodium chloride (NaCl), and potassium chloride (KCl). Three hours were spent defining the release profile.

Five-milliliter aliquots of PBS containing the released drug were taken out at predefined intervals (0.0, 0.25, 0.5, 1.0, 1.5, 2.0, 2.5, and 3.0 h). After each withdrawal, a newly prepared buffer solution was added to maintain the sink conditions and a volume of 250 mL. After filteration through 0.45 µm membrane filter, the samples were examined at 260 nm in a double-beam UV-visible spectrophotometer. PBS dispersion of plain nanoemulsion without PSZ was used as blank in order to avoid the interference with the component of oil.

##### 2.2.7.7 Ulcer index assessment

###### 2.2.7.7.1 Animal handling

Male albino rats were used to calculate the *in-vivo* ulcer index. The animals, which weighed between 160 and 240 g, were purchased for use in experiments from Cairo Agriculture’s Clinical Laboratory Centre. The animals were housed in a conventional laboratory setting for 7 days at a temperature of 25°C ± 1°C and a relative humidity of 55% ± 5%. There was an abundance of food and water available for the animals. The Institutional Review Board for Animal Research/Studies Animals provided the animal experiment protocols for the next investigations (Approval No. 25-10-2023).

###### 2.2.7.7.2 Preparation of organisms for inoculation in the animal model

*C. albicans* strains were utilized to cause ulcers in the test animals. Standard *C. albicans* strains were culled and suspended in 2 mL of normal saline. Four to five colonies were obtained. A vortex mixer was used to apply external agitation in order to correctly produce the suspension. The cell suspension was then centrifuged for 10 min at a speed of 2,500 × g. Centrifugation was followed by precipitation of the suspended fungus. PBS was used to wash the pelleted cells three times. The cells were then suspended in the buffered solution and counted to determine the final concentration, which was 3 × 108 CFU/mL (Alkhalidi et al., 2020).

###### 2.2.7.7.3 Oral candidiasis development in the rat model and assessment of the created formulation

*Setting up the model with a weakened immune system:* The produced drug-loaded NE formulations were evaluated for efficacy by immunocompromising the experimental animals in accordance with established protocols documented in the literature (Alkhalidi et al., 2018). They were given dexamethasone and tetracycline according to a strict regimen. First, the drinking water of the animals was infused with 0.5 mg/L of dexamethasone and 1 g/L of tetracycline. Following a 7-day course of treatment, the drinking water’s dexamethasone concentration was raised to 1 mg/L and the tetracycline concentration was lowered to 0.1 g/L just 1 day before the animals were infected. Until the conclusion of the trial, this dosage of tetracycline and dexamethasone was maintained.

*Introducing C. albicans strains into animals with impaired immune systems:* The absence of any previous *C. albicans* infection in the oral cavity served as the exclusion criterion during the initial examination of the immunocompromised animals. Using the prepared fungal suspension (0.1 mL) on a cotton swab, the animals that were not infected with *C. albicans* were infected three times on days 3, 5, and 7 over their whole oral cavity. Lastly, 3 days following the last inoculation, the experimental animals’ swabs were tested for *Candida* infection in the oral cavity and the CFUs were counted.

###### 2.2.7.7.4 Treatment of infected animals

To administer various formulations to the animals, the infected animals were split into six groups of three animals each. The animals in the first group (U0) were not given any treatment and were considered a control group; the second group (U1) was treated with the PSZ aqueous dispersion; the third group (U2) received the optimized PSZ-CO-CS NE; the fourth group (U3) received the optimized formulation generated without CS; the fifth group (U4) received the optimized formulation prepared without PSZ; and the final group (U5) received the optimized NE created using oleic acid instead of CO. After receiving the treatments for 3 days in a row, the animals’ ulcer indices were checked; these were scored as indicated in Table 2 and the results were analyzed appropriately.

**TABLE 2:** Scoring of the oral ulcers resulting from infection with *C. albicans* (Alkhalidi et al., 2020).

Condition	Score
Ulcers >2 mm	5
Ulcers between 1 and 2 mm with hemorrhagic streaks	4
Ulcers between 1 and 2 mm without hemorrhagic streaks	3
Spot ulceration less than 1 mm	2
Red coloration	1
Normal-colored epithelial lining	0

## 3 Results and discussion

### 3.1 Solubility study of PSZ in different surfactants and co-surfactants

Regarding PSZ’s solubility in surfactants, the results indicated that the active agent acquired the highest solubility in Nonoxynol-10 (25 mg/mL) and the lowest solubility in Polyoxyl 20 cetostearyl ether (7.5 mg/mL). Therefore, Nonoxynol-10 was the surfactant selected to be further employed in the NE fabrication.

**FIGURE 1:**
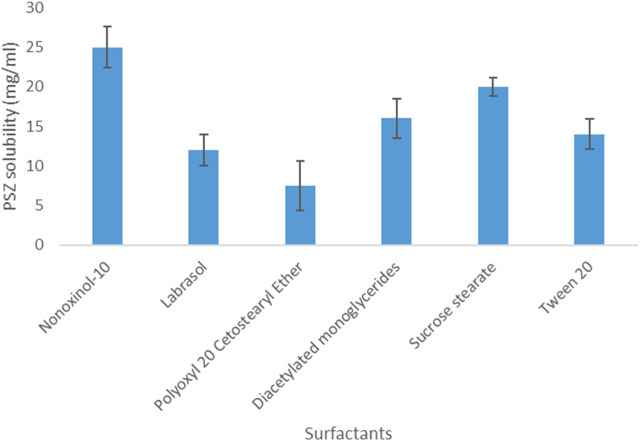
Solubility of PSZ in various surfactants.

**FIGURE 2:**
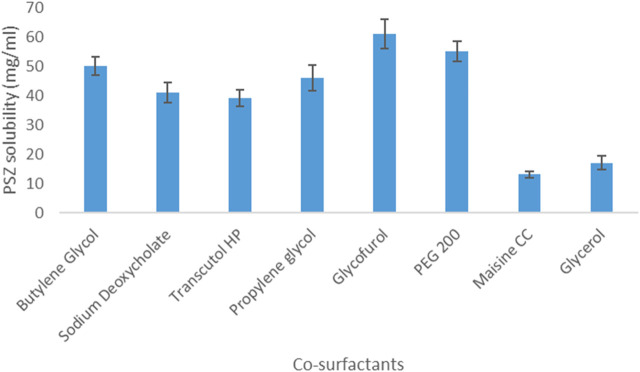
Solubility of PSZ in various co-surfactants.

As for PSZ’s solubility in co-surfactants, the drug was most soluble in glycofurol (61 mg/mL) and least soluble in Maisine CC (13 mg/mL). Hence, glycofurol was the co-surfactant of choice to be incorporated in the manufactured NE formulations. Figures 1, 2 show the solubility of PSZ in various surfactants and co-surfactants.

### 3.2 Pseudoternary phase diagrams

**FIGURE 3:**
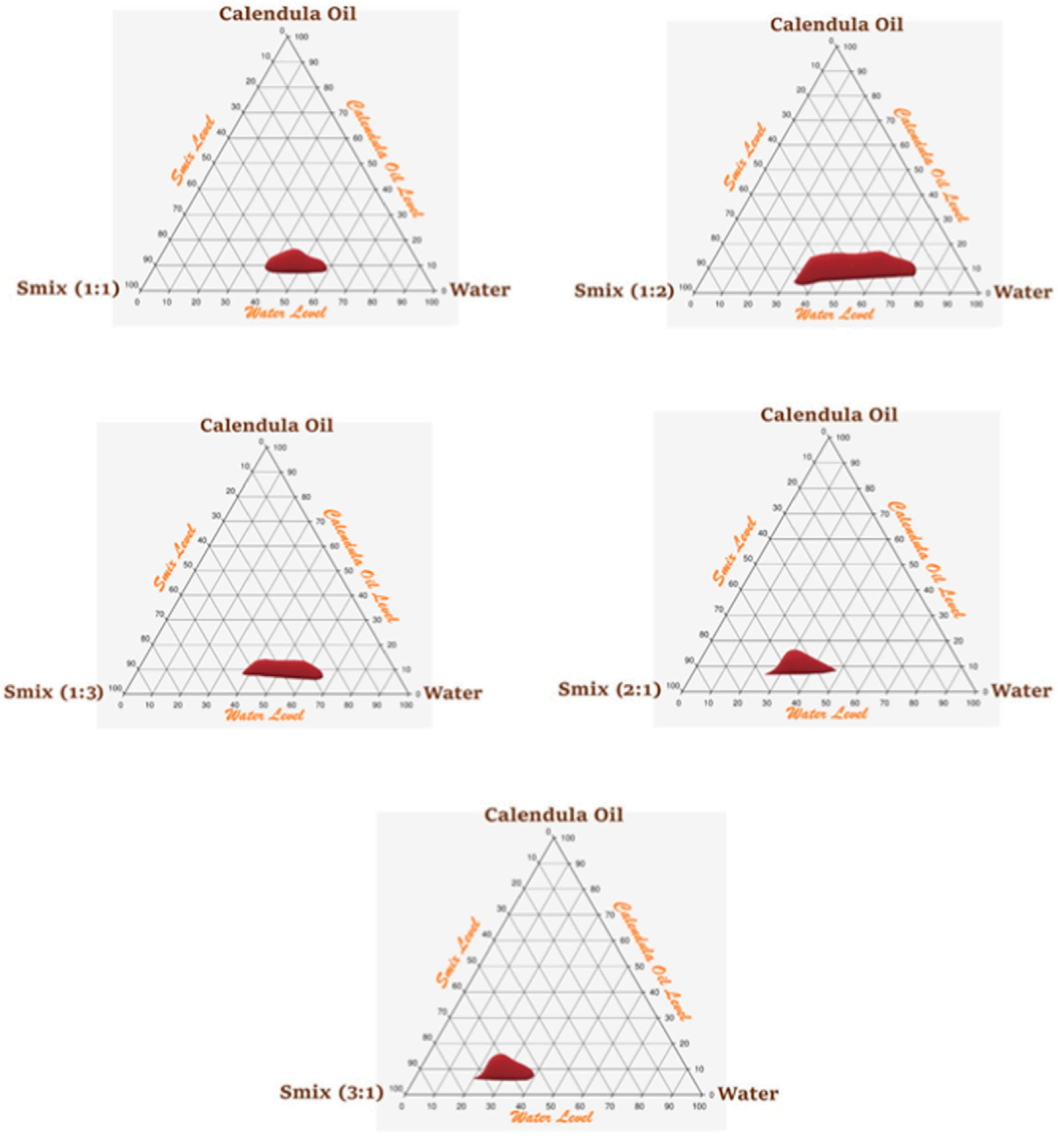
The pseudoternary phase diagrams of CO, the S_mix_, and deionized water.

An S_mix_ ratio of 1:2 of surfactant:co-surfactant was chosen for additional research because it had a wider NE area than the S_mix_ ratio of 1:1, where PSZ was more soluble than the surfactant and the amount of surfactant was suitable for formulating an NE with an oil content of 5%–18%. However, an S_mix_ ratio of 1:3 had a decreased NE region and became thinner (oil content of only 8%–13%) because there was not enough surfactant in the mixture to formulate more than 13% of the CO droplet in a nanosized system. Figure 3 illustrates the pseudoternary phase diagrams.

### 3.3 Characterization of PSZ-CO-CS NE formulations

Initially, it was found that the loading efficiency was >98% for all tested samples if the PSZ used was less than 50 mg/g of nanoemulsion. In our design, the maximum amount of PSZ used in all tested formulations was 20 mg/gm. So, there was no need to incorporate the loading efficiency as one of the dependent variable as it will be more than 98% for all.

#### 3.3.1 Droplet size

The formulations’ appropriate homogeneity, stability, and size distribution were demonstrated by the NE that resulted, with PDI values ranging from 0.09 to 0.32 and droplet sizes ranging from 35 to 150 nm (Table 3).

**TABLE 3:** Box‒Behnken design and responses of PSZ-CO-CS NEs.

Run	A:Amount of posaconazole (mg)	B:Percentage of calendula oil (%)	C:Percentage of chitosan (%)	Y_1_:Vesicle size (nm)	Y_2_:Viscosity (Cp)	Y_3_:Inhibition zone against *Candida* (mm)	Y_4_:Antimicrobial activity against streptococci (mm)	PDI
1	−1	0	1	137	1,200	9	11	0.09
2	1	−1	0	77	730	21	7	0.10
3	0	−1	1	119	1,130	15	6	0.15
4	0	0	0	94	760	18	13	0.17
5	−1	0	−1	67	350	10	12	0.09
6	1	0	−1	76	365	24	16	0.23
7	0	−1	−1	40	290	16	6	0.17
8	0	1	1	148	1,240	17	20	0.30
9	−1	−1	0	81	710	5	3	0.28
10	−1	1	0	99	780	13	18	0.32
11	1	1	0	108	800	25	22	0.11
12	0	0	0	93	765	18	13	0.16
13	0	0	0	95	760	17	14	0.29
14	1	0	1	137	1,210	23	15	0.31
15	0	1	−1	79	360	18	21	0.13
16	−1	−1	−1	35	300	6	4	0.25
17	−1	1	1	141	1,235	12	19	0.19
18	0	0	1	135	1,225	15	15	0.32
19	1	1	1	150	1,260	26	23	0.27
20	1	−1	−1	45	315	22	7	0.18

A quadratic polynomial analysis model was then fitted with the collected globule size data. The explored model was successful in identifying the important effects of the CS percentage (C), CO percentage (B), and PSZ amount (A) boundaries on the droplet sizes of the PSZ-CO-CS NEs, in accordance with the selected mathematical design. Table 4 illustrates the highly connected adjusted R^2^ value of 0.9881 and predicted R^2^ value of 0.9709 for the selected model. Based on an ANOVA data analysis, the following equation was developed:

$$Vesicle\;size=+94.51+2.75\;A+15.50\;B+35.51\;C+2.83\;AB\;‒\;2.67\;AC\;‒\;3.73\;BC+2.05\;A^{2}\;‒\;5.70\;B^{2}+6.84\;C^{2}$$ (3)

**TABLE 4:** Regression analysis results for Y_1,_ Y_2_, Y_3_, and Y_4_ responses.

Dependent variables	R^2^	Adjusted R^2^	Predicted R^2^	F-value	*p*-value	Adequate precision
Y1	0.9937	0.9881	0.9709	175.40	0.0001	38.5683
Y2	0.9997	0.9994	0.9988	3250.7	0.0001	144.7372
Y3	0.9672	0.9611	0.9493	157.43	0.0001	35.3837
Y4	0.9881	0.9859	0.9822	442.79	0.0001	57.2811

**FIGURE 4:**
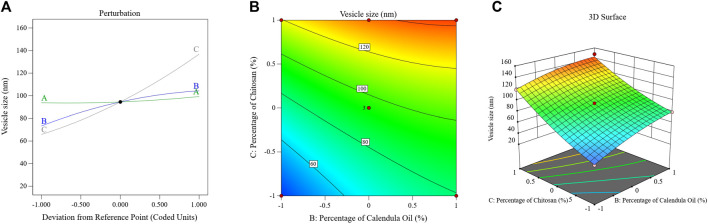
Effects of multiple independent parameters on the droplet sizes of different PSZ-CO-CS NE formulations as shown on perturbation **(A)**, contour **(B)**, and 3D-surface **(C)** plots.

The perturbation, 3D-surface, and contour plots in Figure 4 demonstrate how the parameters impacted the size of the PSZ-CO-CS NE droplets. These figures illustrate how the CS percentages, CO percentages, and PSZ amounts of the formulations influenced the NEs’ final globule sizes.

The above formula and diagram show that all three independent factors positively impacted the NEs’ droplet sizes. As can be seen from the data, the CS (C) and CO (B) affected the size of the droplets (*p* < 0.0001) more significantly than the amount of PSZ (A) (*p* = .03).

Briefly, increasing the quantity of PSZ (factor A) would have been expected to lead to droplet expansion and a bigger emulsion droplet diameter; however, this effect was only marginally observed (*p* = 0.03). Analogous results were observed in the literature (Sakeena et al., 2011; Okur et al., 2020). The correlation between an increase in CO percentage and an increase in droplet size could potentially be accounted for by the fact that a higher oil percentage reduces the levels of the employed S_mix_, which in turn hinders its ability to minimize the droplet size. This produces bigger oil globules, a result that was consistent with findings reported in the literature (Karim et al., 2015). By giving the active agent more space to fit into, increasing the oil content may also have resulted in larger droplets.

The elevated viscosity of the water phase of NE during the emulsification process reduced the resulting net shear stress on the NEs’ organic phase, preventing oil droplet breakup (Mushtaq et al., 2023). This could be the primary reason for the particle size enlargement caused by the increasing CS percentage (factor C). This result could also be explained by a possible physical interaction between CS and the formed oil globules, which is only explained as an interfacial phenomenon. This process increases the amount of adsorbed CS on the surface of droplets, which increases the particle size and leads to the formation of larger emulsion droplets (Jain et al., 2011; Pandit et al., 2017).

#### 3.3.2 Viscosity

Simultaneously, the optimization of the NE viscosity is crucial because it boosts the formulation’s residence time by giving it a sustained release characteristic (Mura et al., 2022). Additionally, an ideal viscosity is needed for easier topical application and better retention at the wound site (Mura et al., 2022). The resulting NE exhibited viscosity values oscillating from 290 to 1,260 cP (Table 3). The secured data showed that most of the formulations acquired a viscosity suitable for application.

The viscosity data that were gathered were then used to fit a quadratic polynomial analysis model. According to the chosen mathematical design, the investigated model was successful in determining the significant effects of the PSZ amount (A), the CO percentage (B), and the CS percentage (C) limits on the viscosity of the PSZ-CO-CS NE. Table 4 shows the predicted R^2^ value of 0.9988 and the highly related adjusted R^2^ value of 0.9994 for the chosen model, signifying model validity. The following formula was created using an ANOVA data analysis:

$$\text{Viscosity}=+764.17+8.75\;A+40.19\;B+429.44\;C+1.25\text{ AB}+0.0000\text{ AC}+11.01\text{ BC}+7.60\;A^{2}‒\;18.65\;B^{2}+12.36\;C^{2}.$$ (4)

**FIGURE 5:**
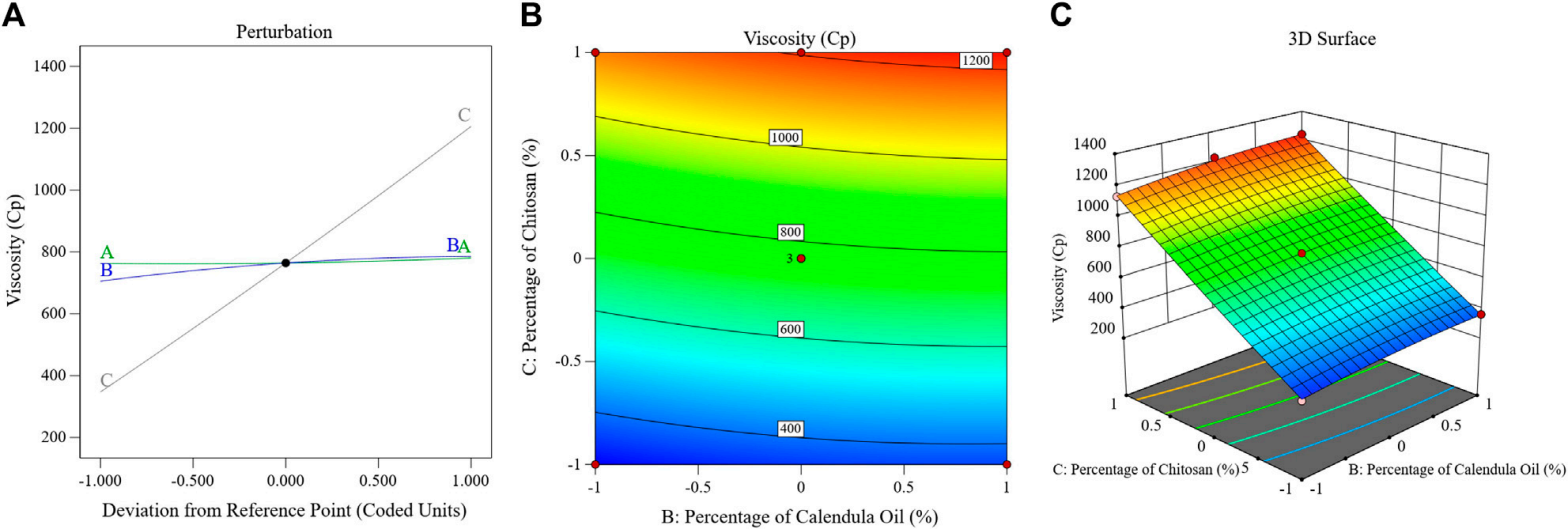
Impact of multiple independent parameters on the Tur loading capacity in different PSZ-CO-CS NE formulations using perturbation **(A)**, contour **(B)**, and 3D-surface **(C)** plots.

The influence of the factors on the viscosity of the PSZ-CO-CS NE is illustrated in Figure 5 through the perturbation, 3D-surface, and contour plots. These data show how the formulations’ CS percentages, CO percentages, and PSZ amounts affected the final viscosity of the NEs.

The mathematical equation and figure above demonstrate how the viscosity of the NEs was positively impacted by each of the three independent parameters. The presented data clearly show that factor A, or the quantity of PSZ (*p* = .008), had a less significant impact on the viscosity than did the percentage of CS (factor C) or percentage of CO (factor B) (*p* < 0.0001). In summary, a more viscous emulsion would have resulted from increasing the PSZ quantity (factor A); however, this effect was only weakly detected (*p* = 0.008). Similar outcomes were noted in previous research (Li et al., 2023).

Factor B (CO percentage) had a more significant positive influence on the NEs’ viscosity (*p* < 0.0001), and this implied that when the oil concentration in the produced NEs was raised, the viscosity of these NEs increased comparably. Such an enhancement in viscosity could be ascribed to the expected decrease in the amount of S_mix_ accompanying the percentage of increase in oil. Consequently, the ability of the surfactant blend to reduce the interfacial tension between the aqueous and organic phases would decrease, leading to larger oil droplets and, hence, greater viscosity. Similar outcomes were reported previously (Massarweh and Abushaikha, 2020).

It was also noteworthy that upon increasing the CS percentage in the developed formulations, the viscosity of these formulations also increased. Such boosting of the formulations’ viscosity is understandable in the light of the CS gelation mechanism in which CS becomes cross-linked with another polymeric chain of its own to generate the most basic type of chemical hydrogel (Ahmadi et al., 2015).

#### 3.3.3 Antifungal efficiency of the various PSZ-CO-CS NE formulations

The inhibition zones produced by the NE formulations against *C, albicans* were oscillated at 5–26 mm, as shown in Table 3. The obtained inhibition zone data were fitted using a unique polynomial analysis linear model. Using the chosen statistical model, the growth zone inhibition capacities of the PSZ-CO-CS NE formulations were analyzed to evaluate the main effects of the CS percentage (C), CO percentage (B), and PSZ amount (A). The selected model achieved an adjusted R^2^ value of 0.9611 and a predicted R^2^ value of 0.9493, as indicated in Table 4. The adjusted and predicted R2 values were found to be closely correlated. The following equation was elaborated by an ANOVA analysis of the collected data:

Inhibition zone against Candida=+16.52+7.17 A+2.29 B ‒ 0.3587 C (5)

**FIGURE 6:**
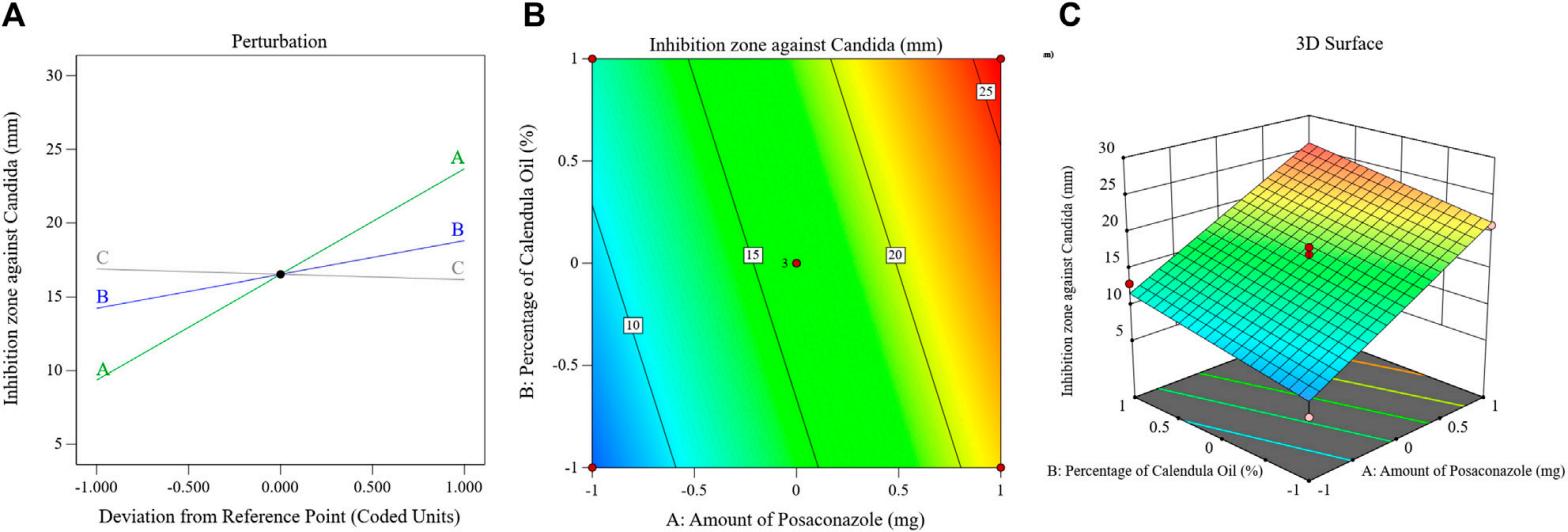
Effects of different parameters of the fungal growth inhibition zones of different PSZ-CO-CS NE formulations. The perturbation **(A)**, contour **(B)**, and 3D-surface **(C)** plots are shown.

The major effect, contour, and 3D-surface graphs showing how the studied parameters affected the PSZ-CO-CS NE formulations’ fungal growth inhibition zones against *C. albicans* are seen in Figure 6.

The graph and equation above suggest that the PSZ and CO had a significant ability to increase the growth inhibition zones of *C. albicans*, whereas the CS did not affect the antifungal efficiency of the developed NEs (*p* = 0.3).

Ergosterol is a unique type of sterol that is exclusively found in the outer membranes of fungi and is essential to the proper growth and functioning of these organisms. Therefore, compounds that influence its level may also have antifungal qualities (Rodrigues, 2018). The main components of CO, sesquiterpene hydrocarbons, epi-α-muurolol, δ-cadinene, and α-cadinol, were found to have the ability to inhibit fungal growth by altering the way fatty acids, particularly ergosterol, were metabolized in these cells (Gazim et al., 2008).

As illustrated by the preceding figure and mathematical formula, PSZ attained the most profound antifungal activity amongst the developed NEs’ components. Like the other triazole antifungals, PSZ inhibits the fungal enzyme lanosterol 14-alpha-demethylase (Herbrecht, 2004; Torres et al., 2005). A reduction in this enzyme causes a decrease in fungal ergosterol synthesis, which is vital for the formation of fungal cell walls. The cell wall abnormalities result in either cell death or blunted cell growth (Sridhar et al., 2003).

#### 3.3.4 Antibacterial activities of PSZ-CO-CS NEs

Table 3 displays the inhibitory zones of the NE formulations against streptococci, which were exposed to oscillations of between 4 and 23 mm. A special linear model for polynomial analysis was used to fit the zone inhibition data that were acquired. The growth zone inhibition capacities of the PSZ-CO-CS NE formulations were examined using the selected statistical model in order to assess the primary impacts of the CS percentage (C), CO percentage (B), and PSZ amount (A). Table 4 shows that the chosen model had an adjusted R^2^ value of 0.9859 and a predicted R^2^ value of 0.9822, which were closely related and suggested model validity. An ANOVA analysis of the gathered data resulted in the formulation of the following equation:

Antimicrobial activity against streptococci=+13.25+1.92A+7.51 B ‒ 0.0215 C (6)

**FIGURE 7:**
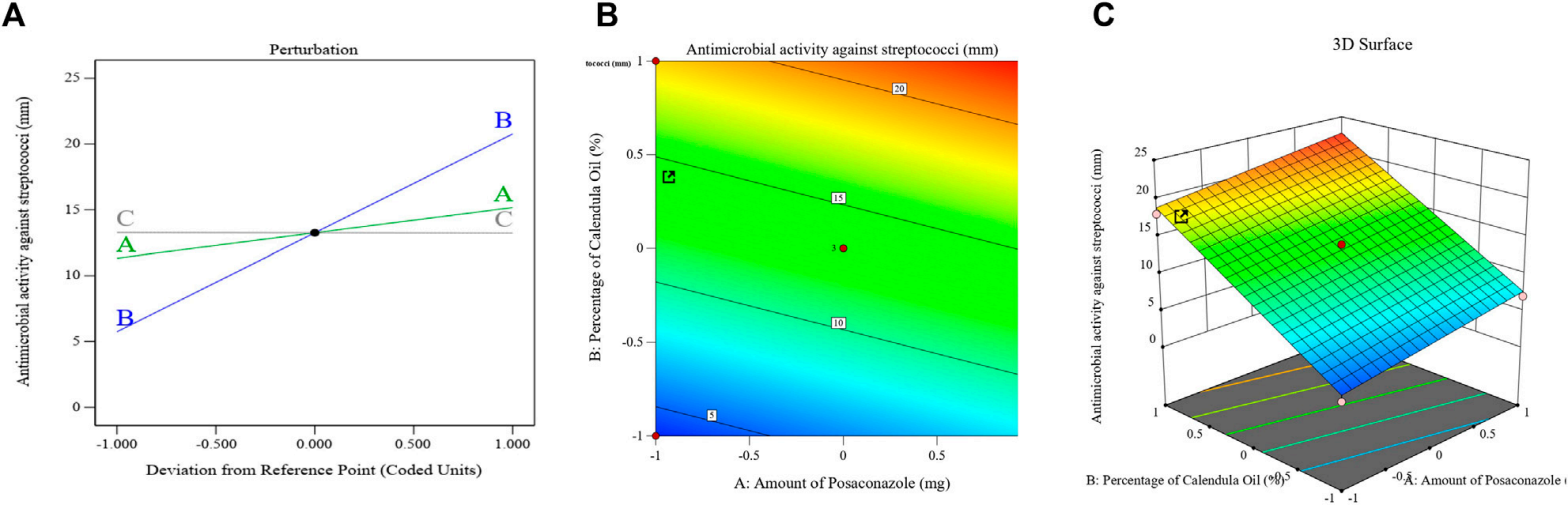
Effects of different parameters that have been explored on the bacterial growth inhibition zones of different PSZ-CO-CS NE formulations. The perturbation **(A)**, contour **(B)**, and 3D-surface **(C)** plots are shown.

Figure 7 displays the principal effect, contour, and 3D-surface graphs that demonstrate how the parameters under study affected the bacterial growth inhibition zones of streptococci in the PSZ-CO-CS NE formulations.

The above equation and graph show that the CO exhibited the most antibacterial activity amongst the employed NE constituents (*p* < 0.0001).

The bacterial cell membranes were deformed and were more permeable due to the high concentration of sesquiterpenes in the CO, which function as phytoalexins, antibiotic substances that can intercalate in the lipids of the bacterial cell membrane (Devi et al., 2010). The anticancer, anti-inflammatory, and antibacterial properties of this class of drugs have also been described by Salazar-Gómez et al. (2020) (Salazar-Gómez et al., 2020). Major components of an essential oil can be responsible for its antibacterial action, even though the effect is dependent on the composition of metabolites in the oil (Ruíz-Posadas et al., 2023).

Further, calendula essential oil contains a variety of compounds, including caryophyllene, which has been shown to be effective against *S. aureus* (De Lima et al., 2010); cubebene, which inhibits the growth of *S. aureus* and *Pseudomonas aeruginosa* (Sharifi-Rad et al., 2014); and cadinene (Salomé-Abarca et al., 2015), which has anti-inflammatory and antiseptic characteristics (Kaufman et al., 1999).

PSZ was also found to contribute to the antibacterial effect of the suggested formulations; however, it exerted such effects to a lesser extent.

The enzyme flavohemoglobin (flavoHb), a dioxygenase, is responsible for the antibacterial properties of azoles such as PSZ. Such an enzyme transfers electrons between NAD(P)H, FAD, heme, and oxygen, the natural substrate, to convert nitric oxide to nitrate (El Hammi et al., 2012). As one aspect of its defense mechanism against host insults, flavoHbs are known to shield bacteria from nitrosative stress (Frey et al., 2002). Here, azole antifungals were discovered to attach to the catalytic site of this enzyme through interactions with the enzyme’s heme cofactor, disrupting the bacterial cell growth. It is worth mentioning that CS (factor C) exerted no significant impact on the antibacterial properties of the developed NEs.

### 3.4 Optimization of the characterized PSZ-CO-CS NEs

**FIGURE 8:**
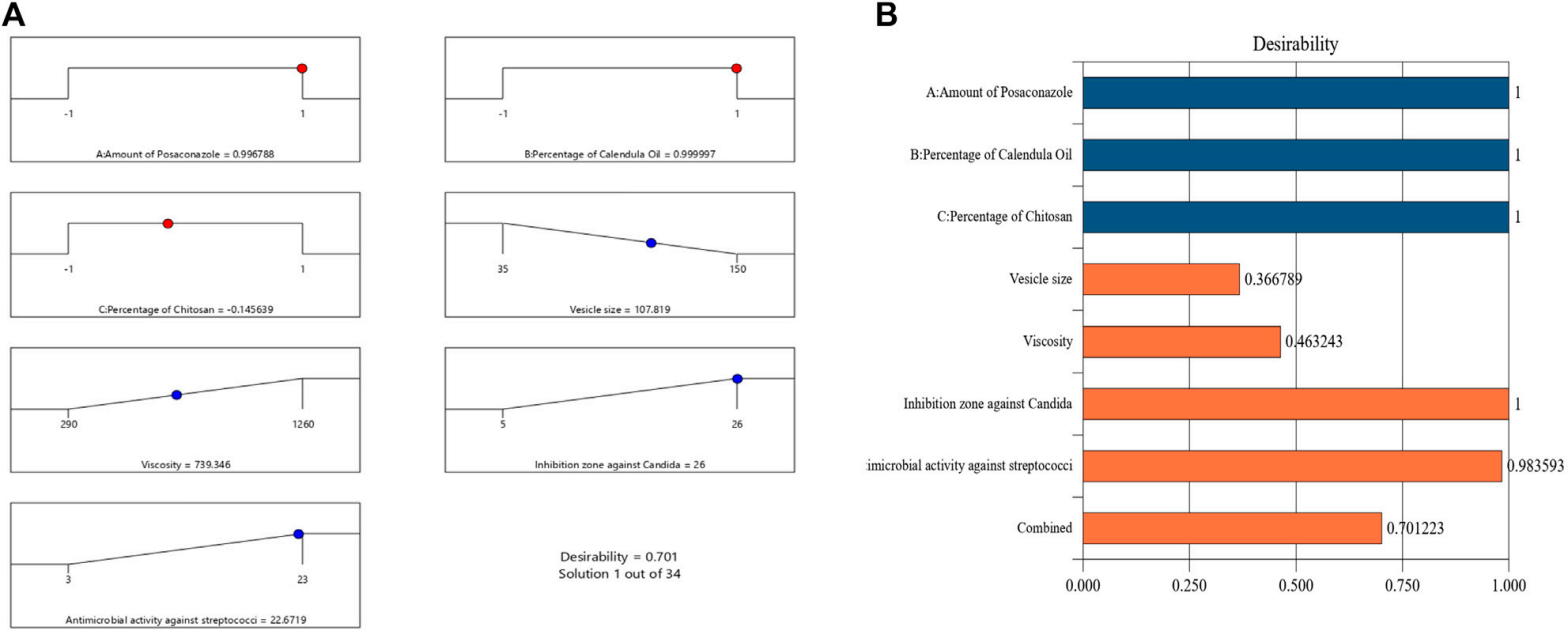
Bar chart and desirability ramp for optimization. **(A)** The levels of independent variables and anticipated values for the responses of the best formulation are displayed on the desirability ramp. **(B)** The aggregated responses’ desirability values are displayed in a bar chart.

An NE formulation with the best specifications—that is, the ideal formulation—was established once the tests outlined were finished. The experimental design recommended different sets of independent variables. The ideal formulation contained 18% of CO, 20 mg of PSZ, and 1.35% of CS, with a desirability value of 0.701. The ideal PSZ-CO-CS NE had a mean droplet size of 110 nm, viscosity of 750 cP, *C. albicans* inhibition zone of 25 mm, and inhibition zone against streptococci of 22 mm. These outcomes closely matched the predicted values of the identical responses, which were a mean droplet size of 107 nm, viscosity of 739.3 cP, *C. albicans* inhibition zone of 26 mm, and inhibition zone against streptococci of 22.6 mm. Additionally, the optimum formulation attained a zeta potential value of 28.3 mV and a PDI of 0.208. The desirability ramp and bar chart for the various levels of the elements under study and the anticipated dependent variables of the ideal formulation are made clearer in Figure 8. It was noteworthy that there is augmentation in the design and this was optional in the software used in the analysis, this augmentation of the design is suggested by the software during building of the experiment. This may consume more time and material but in same time make the results more accurate and the optimum formula give observed values nearly equal more to the predicted values.

### 3.5 Characterization of the fabricated optimal PSZ-CO-CS NE formulation

#### 3.5.1 Antifungal studies for the optimized PSZ-CO-CS NE formulation

The collected data revealed that formulation F1, the optimized PSZ-CO-CS NE, acquired a growth inhibition zone of *C. albicans* of 25 mm, while formulation F2, the optimized formulation prepared without PSZ, exhibited the lowest value for zone inhibition of 8 mm. The optimized formulation prepared with oleic acid instead of CO, F3 and the oily dispersion of PSZ in CO, F4, had a zone of inhibition of *C. albicans* of 18 mm and 16 mm, respectively. These results revealed the paramount importance of PSZ in the formulations’ antifungal efficiency, since its absence resulted in a dramatic decrease in the diameter of the *C. albicans* zone of inhibition. Moreover, CO is thought to contribute considerably to the antifungal characteristics of the developed optimal formulation as the preceding results illustrated that replacing CO with oleic acid yielded a much narrower *C. albicans* zone of inhibition (18 mm) compared with that of the optimal formulation (25 mm). The nanosized drug delivery system appeared also to significantly influence the antifungal properties of the optimal formulation. This was affirmed by the results for formulation F4, the oily dispersion of PSZ in CO, which had a small inhibition zone of *C. albicans* growth (16 mm) compared with that of the optimal formulation based on a nanosized drug delivery system.

#### 3.5.2 Microbiological activities of the optimized PSZ-CO-CS NE formulation

The zones of inhibition for the tested optimized formulation PSZ-CO-CS NE (F1), the optimized formulation made without PSZ (F2), the optimized formulation made with oleic acid in place of CO (F3), and the oily dispersion of PSZ in CO (F4) were identified during this experiment.

Values of 22, 16, 6, and 14 mm were obtained for formulations F1, F2, F3, and F4, respectively.

The above results uncovered the vital role of CO in the antibacterial activity of the optimal formulation. As might be noticed, the absence of CO in formulation F3 (which contained oleic acid in place of CO) yielded a much smaller growth inhibition zone of 6 mm compared with the zone of 22 mm in formulation F1. It is also interesting to mention the antibacterial role that might be associated with the CS content of the optimal formulation. Chitosan’s antibacterial activities are attributed to its polycationic character, which facilitates its liaison with negatively-charged bacterial cell envelopes and cytoplasmic membranes. A reduced osmotic stability, more frequent membrane rupturing, and, ultimately, the leakage of intracellular elements are the outcomes of these interactions (Yilmaz Atay, 2020).

The above results also show that PSZ played a key role in the antibacterial activity of the NEs. However, this role is considered somewhat minor compared with the role of CO, as the absence of PSZ, as in F2, reduced the growth inhibition zone to 16 mm compared with the 22 mm of the optimal formulation (F1).

The nanoscale drug delivery technology seems to have contributed significantly to the antibacterial characteristics of the best formulation. This was shown by the results of formulation F4, which produced a smaller inhibition zone of streptococcal growth (14 mm) than the ideal formulation, which was based on a nanoscale drug delivery system. In this formulation, the PSZ was spatially distributed throughout the CO.

#### 3.5.3 Stability using the freeze‒thaw cycle

The stability index calculated for the optimal formulation was 86%. This indicated an acceptable stability of the developed formulation (Hosny et al., 2021b). These results were harmonious with the particle size results, which confirmed the homogeneity and physical stability of the small nanoglobules of oil in the NE, which in turn ensured the continuous suspension of oil globules in the continuous outer phase, thus lowering the chance of sedimentation (Hosny et al., 2021b).

#### 3.5.4 Rheologic characteristics of the PSZ-CO-CS NE formulations

**FIGURE 9:**
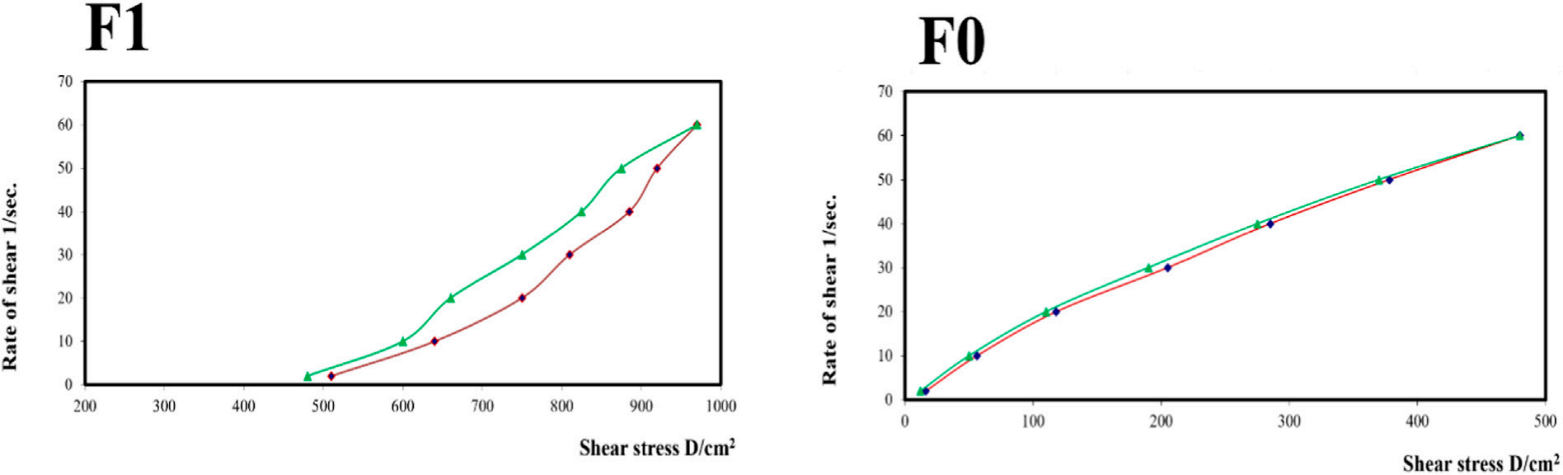
Rheograms of the different tested formulations.

**FIGURE 10:**
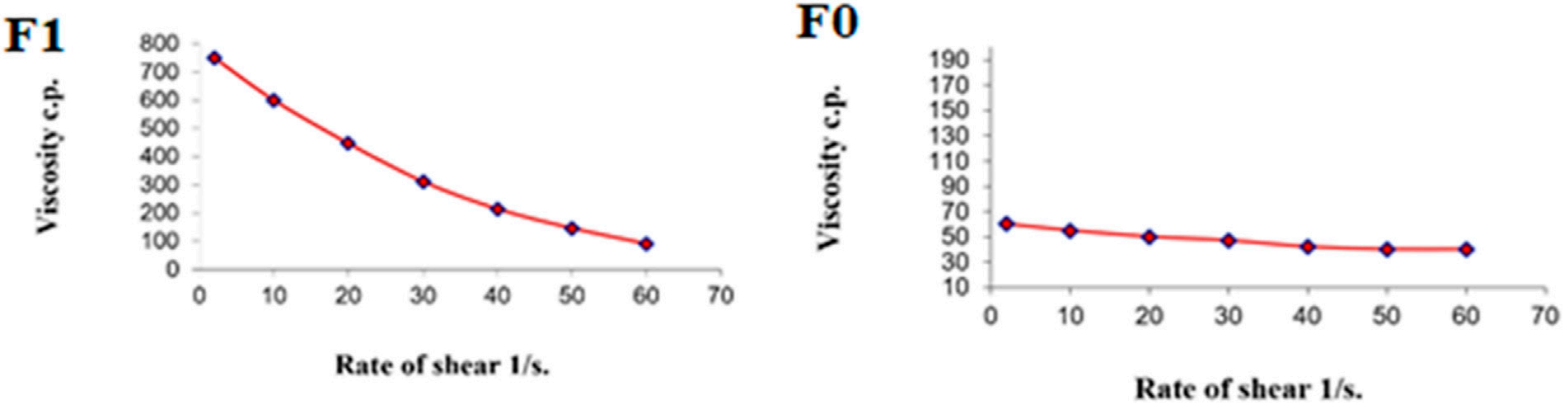
Representative plots of the rate of shear (G) *versus* the viscosity (η) for the tested formulations.

**FIGURE 11:**
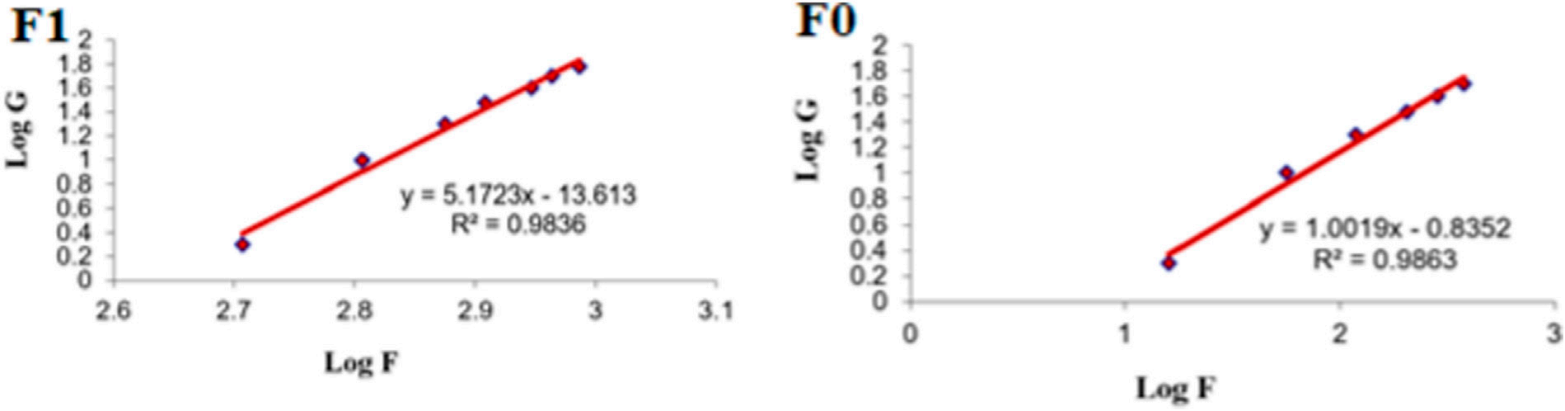
Representative plots of the logarithm of the rate of shear (G) *versus* the logarithm of the shearing stress (F) for different formulations.

Figure 9 shows the rheograms for the tested formulations. It was clear that for formulation F1, which contained CS, there was a discernible increase in the rate of shear with the increase in shear stress, in contrast to formulation F0, which did not contain CS, and the flow of the latter formulation was mainly Newtonian. Further, Figure 10, which illustrates the plot of the viscosity against the rate of sheer, reveals that the viscosity was greatly reduced when the shear rate was increased in formulation F1, while quite the opposite was observed in the case of formulation F0, in which the viscosity remained almost constant with a changing rate of sheer. Such results could also be ascribed to the presence of CS in F1 and its absence in F0. Additionally, Farrow’s constant was obtained from plots representing the log rate of the shear against the log shear stress (Figure 11). The constant values were found to be 5.172 and 1.009 for formulations F1 and F0, respectively. These outcomes proved the pseudoplastic flow of F1 and the Newtonian flow of F0. The F1 formulation demonstrated thixotropic behavior with pseudoplastic flow, a desired property for topical medication formulations. This flow characteristic of formulations containing CS could be the consequence of shear, which breaks down the structural arrangement by rupturing the intermolecular bonds between polymeric chains (Lewandowska and Szulc, 2021).

#### 3.5.5 *In vitro* release of the PSZ-CO-CS NE formulations

**FIGURE 12:**
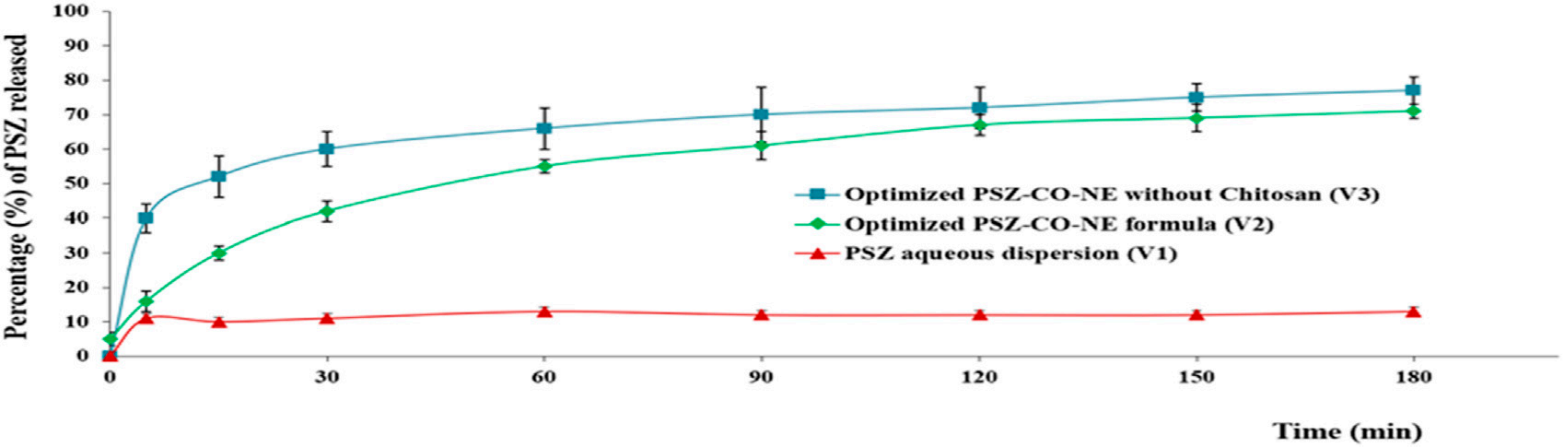
*In vitro* release behavior of PSZ from different PSZ-loaded formulations.

As is obvious from the above curve, Figure 12 PSZ was the lowest from formulation V1, the PSZ aqueous dispersion, at 13%, while the highest release percentage of the drug was from formulation V3, which encompassed the optimal PSZ-CO NE prepared with no CS, at 77%. The release percentage of PSZ from formulation V2, the optimal PSZ-CO-CS NE, which contained 135% CS (73%), was lower than that from formulation V3. However, such release behavior was found to be more consistent and more controlled and had lower SD values. The slightly lower percentage of PSZ from V2 could be due to the presence of CS, which made the formulation more viscous and, hence, hindered the release of the drug from the NE. Nonetheless, it also gave the drug release a more controlled pattern. Similar outcomes were formerly reported in the literature (Malik et al., 2022).

#### 3.5.6 Ulcer index

Table 5 shows that the group that received the optimal formulation had the lowest ulcer index (0.5 ± 0.5); this implied that only a red coloration of the gingiva was observed. The group that received the PSZ aqueous dispersion had the second highest ulcer index (4.0 ± 1.0), and the control group had the highest value (4.5 ± 0.5). The results for the latter two formulations revealed the presence of ulcers of 1–2 mm with hemorrhagic streaks.

**TABLE 5:** *In vivo* ulcer index of the *C. albicans*‒infected immunocompromised animals after 3 days of treatment.

Formulation	Ulcer index
U0	4.5 ± 0.5
U1	4.0 ± 1.0
U2	0.5 ± 0.5
U3	1.5 ± 0.5
U4	2.5 ± 0.5
U5	2 ± 1

*Data are expressed as mean ± SD (n = 3).

The outstanding performance of the optimal PSZ-CO-CS NE in treating buccal ulcers might have been due to its components. For instance, CO appeared to contribute to the ulcer-healing properties of the formulation through its profound anti-inflammatory characteristics. It was postulated that CO functions by suppressing COX-2 and pro-inflammatory cytokines (IL-1β, IL-6, TNF-α, and IFN-γ), and the production of prostaglandins that follows, via its inhibition of triggering enzymes (Preethi et al., 2009). Moreover, PSZ was reported to have a secondary anti-inflammatory potential, and, therefore, its contribution to the anti-inflammatory effect of the NE might be minor (Steel et al., 2009). These postulations could be further affirmed by the rest of the results. For example, the group that received the optimal formulation with no CS (U3) had an ulcer index (1.5 ± 0.5) quite a bit higher than that of group U2, which was treated with the optimal PSZ-CO-CS NE formulation (0.5 ± 0.5). The only difference between them was the absence of CS. Chitosan has been shown to have immune-promoting properties, such as the suppression of pro-inflammatory cytokines, the recruitment of fibroblasts to promote tissue granulation (Friedman et al., 2013), and the synthesis of type III collagen (Ueno et al., 1999). These findings point to the potential applications of CS as an anti-inflammatory and an expedient for ulcer healing.

Moreover, the group treated with the optimal formulation that contained no PSZ (U4) attained an ulcer index of 2.5 ± 0.5, implying the presence of ulcers of 1–2 mm without hemorrhagic streaks. Such results might prove that PSZ’s anti-inflammatory action could be somewhat weak. In addition, the group treated with the optimal formulation containing oleic acid instead of CO as an oil phase had an ulcer index of 2 ± 1, which indicated the presence of a spot ulceration of less than 1 mm. Such findings also confirmed the importance of CO in treating gingival ulcers, which could be explained by the previously mentioned conditions (Ruíz-Posadas et al., 2023).

## 4 Conclusion

PSZ was effectively produced as a functional NE along with incorporating CO and CS. A pseudoternary phase diagram was used to determine the ideal level of S_mix_ for the necessary medication delivery system. The created NEs showed a suitable homogeneous distribution, with globule sizes below 200 nm, and a PDI of less than 0.35 indicated that the systems were sufficiently stable. The response surface Box‒Behnken design was used to generate the ideal formulation, which had 1.35% of CS, 20 mg of PSZ, and 18% of CO. Additionally, the optimum formulation was able to produce fungal and bacterial growth inhibition zones of up to 25 and 22 mm, respectively, a particle size of 110 nm, and a viscosity of 750 cP. After that, the optimal formulation showed an enhanced and controlled drug release. The optimized PSZ-CO-CS NE demonstrated the widest bacterial and fungal growth inhibition zone, reasonable rheologic characteristics, and the lowest ulcer index in rats when compared with the other tested formulations. Overall, this study showed that formulations containing PSZ and CO-based and CS-decorated NEs had the ability to effectively manage gingivitis.

## Data Availability

The original contributions presented in the study are included in the article/supplementary material, further inquiries can be directed to the corresponding author.
